# Cardiovascular disease occurrence in two close but different social environments

**DOI:** 10.1186/1476-072X-10-5

**Published:** 2011-01-12

**Authors:** Carina Wennerholm, Björn Grip, Annakarin Johansson, Hans Nilsson, Marja-Liisa Honkasalo, Tomas Faresjö

**Affiliations:** 1 Department of Medicine and Health Sciences, Linköping University, SE-581 83 Linköping, Sweden; 2 Department of Studies of Social Change and Culture, Linköping University, SE-581 83 Linköping, Sweden

## Abstract

**Background:**

Cardiovascular diseases estimate to be the leading cause of death and loss of disability-adjusted life years globally. Conventional risk factors for cardiovascular diseases only partly account for the social gradient. The purpose of this study was to compare the occurrence of the most frequent cardiovascular diseases and cardiovascular mortality in two close cities, the Twin cities.

**Methods:**

We focused on the total population in two neighbour and equally sized cities with a population of around 135 000 inhabitants each. These twin cities represent two different social environments in the same Swedish county. According to their social history they could be labelled a "blue-collar" and a "white-collar" city. Morbidity data for the two cities was derived from an administrative health care register based on medical records assigned by the physicians at both hospitals and primary care. The morbidity data presented are cumulative incidence rates and the data on mortality for ischemic heart diseases is based on official Swedish statistics.

**Results:**

The cumulative incidence of different cardiovascular diagnoses for younger and also elderly men and women revealed significantly differences for studied cardiovascular diagnoses. The occurrence rates were in all aspects highest in the population of the "blue-collar" twin city for both sexes.

**Conclusions:**

This study revealed that there are significant differences in risk for cardiovascular morbidity and mortality between the populations in the studied different social environments. These differences seem to be profound and stable over time and thereby give implication for public health policy to initiate a community intervention program in the "blue-collar" twin city.

## Background

In Sweden mortality from cardiovascular diseases (CVD) gradually increased from the beginning of the 20^th ^century until the 1960s, when this trend reached a plateau during the 1970s and thereafter in the beginning of the 1980s decreased for both men and women with 50% the last two decades in Sweden as in other industrialised countries [1,2]. This decline in cardiovascular mortality could be explained by two factors: the risk of developing heart infarction has decreased due to better lifestyle and the chance of surviving a heart attack has increased [3-5]. Both incidence of and mortality due to CHD are today significantly higher in subjects with low socio-economic status [6]. In many industrial countries here is a clear trend of widening social class differences. Increased socio-economic differences of the risk for myocardial infarction have been reported last decade in Sweden [7].

Conventional risk factors for cardiovascular diseases (CVD) such as high blood pressure and smoking only partly account for the social gradient for CVD that has been widely demonstrated [6,8]. Studies in developed countries show that low income is associated with higher incidence of coronary heart disease and higher mortality after a heart attack [9]. The socio-economic differences in Sweden for cardiovascular disease are still high but compared to fifteen years ago the social differences in terms of educational level and CVD risks are slightly less [10].

Like most western societies, Sweden, is becoming a more multicultural society with a mix of natives and first or second-generation immigrants, which also has implications for the occurrence of cardiovascular diseases in the population. A Swedish study has found that immigrants have an increased incidence of acute myocardial infarction compared to natives. Immigrants also have a higher incidence of myocardial infarction including non-fatal as well as fatal out-of-hospital cases, than Swedish born people after adjustment for age and socio-economic group [11].

To better understand disease occurrence, treatment, health outcomes, and prevention it has for centuries been evident in medicine to also include different social determinants [12]. The role and importance of social and physical environment for health, the effect of place, and the question of whether we should focus on places or people is still a relevant matter for public health science [13-16]. A general conclusion of this issue is that who you are and how you live your life as well as the place where you live your life are of importance for health [17]. By tradition there are two perspectives for studying determinants of health in public health and social medicine; one focuses on "upstream" factors and the other on "downstream" factors. The emphasis in the upstream approach is on social, ethnical, cultural and economical factors in the community. According to this perspective, individual differences in lifestyles and living conditions are not the only important factors in explaining health differences between individuals; social, ethnical, cultural and economic factors in the community will also affect public health and generate health differences between individuals [18,19]. A phenomenon noticed is that defined populations in one geographical area could display a particular distribution of risk factors that differ markedly from those found in a similar population but in another geographical area [20,21]. The downstream perspective, which is predominant in health care research and clinical practice, focuses on the individual and on individual living conditions and lifestyles. According to this downstream perspective disease occurrence in an individual must either be related to environmental exposure or genetically inherited. However, in public health research, it is not unusual to combine these two perspectives [22,23].

Neighbourhood of residence has been suggested to affect cardiovascular risk above and beyond personal socio-economic status [24,25]. In a multilevel analysis it was found that neighbourhood income predicted individual systolic blood pressure, but it was concluded that both individual and neighbourhood socio-economic status and race were linked to cardiovascular risk disparities as early as adolescence [26,27]. A Swedish study has reported that structural economic conditions in neighbourhoods do matter with regard to adult social class inequalities [28]. The effect is not restricted to lower social strata, but as individuals in lower social strata more often live in disadvantaged contexts, and also seem to be more vulnerable to the effects of these contexts, it was concluded that economic segregation creates neighbourhood contexts that contribute to social class inequality in incidence of myocardial infarction [29].

In order to address an upstream perspective of cardiovascular disease occurrence in the population we conducted a study focusing on the population in two comparable and equally sized Swedish cities, the Twin cities.

### Study aims

The aim of this study was to compare the occurrence of the most frequent cardiovascular diseases and cardiovascular mortality in two close but different social environments, the Twin cities.

## Methods

### Study population

A description of some indicators underpinning the differences in social environments of the two twin cities is given in table 1. The two cities a "blue-collar" city (Norrköping) and a "white-collar" city (Linköping) are located only 25 miles apart in the same county in the southeast of Sweden. Both cities are served by the same health care organization; the same County Council is responsible for all public-funded health care in the two cities. The private health care sector is only a marginal phenomenon in the region. At the time of the study, each of the cities had around 135 000 inhabitants and almost the same age distribution.

**Table 1:** Indicators of the social environment in the "white-collar" and "blue-collar" twin city.

		White-collar twin city	Blue-collar twin city
Total number of inhabitants (2009)		144 690	129 254

Life expectancy at birth (2006)	Women	82.6 years	81.8 years

	Men	79.5 years	77.9 years

Inhabitants born outside Europe (2006)		9.6%	11.0%

Inhabitants aged 20-64 years with high income (2006)#		21.2%	16.9%

Inhabitants aged 20-64 years with high educational level (2006)##		45.9%	30.7%

Crime of violence per 100 000 inhabitants (2009)		976	1506

Economic social aid SEK/inhabitants in the city (2009)		1391 SEK	1715 SEK

Unemployment rate aged 20-64 years (2009)	Women	3.0%	4.0%

	Men	4.3%	5.5%

Participation rate in the National election (2006)		85.0%	81.5%

Note: Data for 2006 from Official Statistics, the Swedish Institute of Public Health and Statistics, Sweden.

#High income refers to the 20% of the inhabitants nationally with the highest income. ## High education refers to 12 or more years of education.

Today the cities are quite similar but their social history differs. We therefore generalize the urban identity of the cities based on their history. The term "white-collar" is generally used to refer to work that does not require strenuous physical labor. The term "blue-collar", conversely, refers to workers whose work requires manual labor. We use the terms to describe the two different social environments and geographical areas. Manual laborers mostly inhabit a "blue-collar" community and a "white-collar" community contains mostly administrators.

During the 17th century, the "blue-collar" twin city received its rights to foreign trade and an international port. This was the beginning of a development that made the "blue-collar" twin city a centre for textile industry, "The Manchester of Sweden". Over time, the textile industry became more mechanised and the number of unskilled workers increased in the "blue-collar" city. Only in recent decades, have non-manual occupations become dominant in "the blue-collar" city and it has also become a university town in the last decade. Meanwhile the "white-collar" twin city was a regional and agricultural centre dominated by a quite life of pupils from the cathedral school. While the industrial revolution went on in the "blue-collar" city the "white-collar" city remained a rural market town, ruled by both the church and the state. For a long time there has also been a significant military presence, with several regiments. After World War II the "white-collar" city grew in terms of population and became industrialized, mainly due to the aviation industry expansion. Today, high technology companies and a university dominate the "white-collar" city.

Although these twin cities today have an extrinsic resemblance in terms of culture, environment and climate, there are tendencies of differences in public health between them which are today manifested in public health indicators such as; life expectancy, prevalence of cardiovascular diseases, sick leave, perceived health and lifestyle factors [30].

There is a long tradition especially in the Scandinavian countries of documenting diseases in registers. In Sweden each inhabitant is assigned a unique personal code based on birth date and gender [31]. By law, the county councils in Sweden are required to report inpatient data to the Swedish Hospital Discharge register on an annual basis, but national registration of outpatient data has not been implemented [31]. These health care databases offer administrative data, but they might also be suitable for population-based epidemiological studies. In one Swedish region (Östergötland County Council) patient data from Primary Health Care (PHC), outpatient hospital care, and hospital care has been recorded for some years in a shared computerized population-based administrative Health Care Register (HCR). We conducted a retrospective register study and used this regional health care database to identify the most common cardiovascular diseases diagnosed and documented in medical records from both hospital care and primary care.

### Data

The administrative Health Care Register (HCR) used in this study is based on computerised data files linked by a unique personal code to birth date and gender of all inhabitants in the region. The same personal code is used for all visits and diagnoses in HCR. The International Classification of Diseases (ICD 10-code) for the CHD disorders presented in this study was: E78 (high cholesterol), I10 (hypertension), I20.9 (angina pectoris), I21 (myocardial infarction), I25 (ischemic heart disease), I50 (cardiac insufficiency), I61.9 (cerebral haemorrhage), I63 (stroke). Physicians in hospitals as well as in primary care assigned these diagnostic codes. The morbidity data presented are cumulative incidence rates. The numerator for the cumulative incidence rates was the number of the first diagnosed cases identified during the six-year study period (2002-2007). The denominator was the number of inhabitants calculated as the mean number in each age group of the population during the six-year study period. The cumulative incidence rates are presented as numbers per 1000 inhabitants.

Swedish national data on mortality for ischemic heart diseases (mainly heart infarction) in the largest Swedish cities (all cities with over or around 100 000 inhabitants) was also included. It represents early mortality in the population from the age of 15 and above in each city between 2002 and 2006 and are age standardised against the national Swedish population [32]. The historical mortality data of cardiovascular diseases in the twin cities is based on national mortality statistics from The Statistics in Sweden.

Data was stored in a shared database and statistically analysed using SPSS 17.0 (SPSS Inc., Chicago, IL, USA). Risk Ratios (RR) and 95% confidence intervals (CI) were calculated based on crude data with the "white-collar" city as the reference group. A p-value of p < 0.05 was considered statistically significant.

## Results

### Cardiovascular mortality

**Figure 1:**
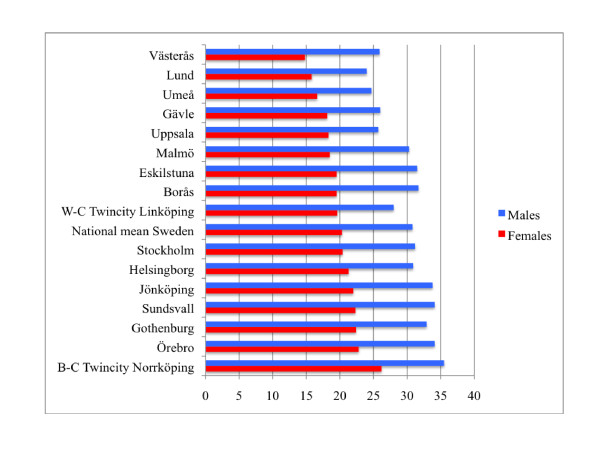
**Mortality in ischemic heart diseases (all ages over 15, age-adjusted) yearly per 10 000 inhabitants for males and females in the 16 largest Swedish cities 2002-2006**. (Data from National Board of Health and Welfare, Sweden; 2010.)

**Figure 2:**
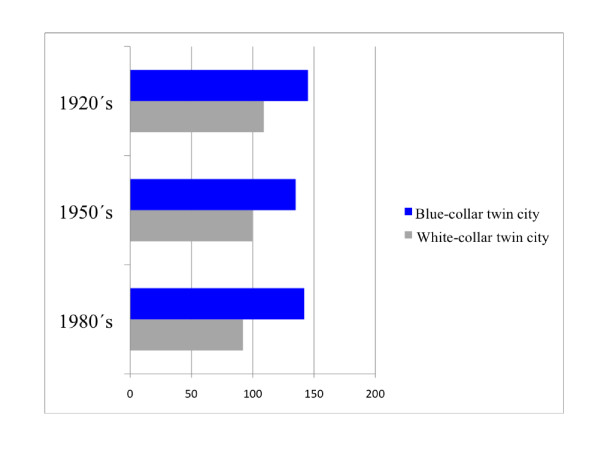
**Historical data of mortality rates from coronary heart diseases in the population of the "white-collar" twin city and the "blue-collar" twin city (Swedish national mean index = 100)**.

Recent Swedish national data (2002-2006) on mortality in connection with ischemic heart diseases in males and females living in the largest Swedish cities are presented in Figure 1. Data from all Swedish cities with around 100 000 inhabitants or more was chosen for this comparison. In general, mortality rates were higher in males than females. The "blue-collar" twin city had the highest mortality rates in connection with ischemic heart diseases in both males and females compared to all other comparable Swedish cities of the same size. In this respect the "white-collar" twin city had lower mortality rates than the national mean in this respect in both females and males. Historical data of cardiovascular mortality for the twin cities is presented in Figure 2. A higher rate of coronary heart mortality is evident for the population in the "blue-collar" twin city from the 1920's until recent decades compared with the "white-collar" twin city.

### Cardiovascular morbidity

The cumulative incidence of different cardiovascular diagnoses in younger men and women (aged 45-64 years) is presented in table 2 and table 3. The comparison between the twin cities revealed significant differences in all studied diagnoses, except for high cholesterol levels. The relative rates were higher for both sexes in the "blue-collar" twin city population.

**Table 2:** Cumulative incidence of cardiovascular diseases and cardiovascular risk factors (according to the diagnoses in ICD-10) and RR (risk ratios) for men aged 45-64 years (diagnosed 2002-2007) in the twin cities.

	White-collar twin city				Blue-collar twin city			
	
	45-64 years (n = 16 057 inhabitants)				45-64 years (n = 16 029 inhabitants)			
	
	n	n/1000	RR	95% CI	n	n/1000	RR	95% CI
High Cholesterol (ICD-E-78)	876	54.6	1.00	-	912	56.9	1.04	(0.95-1.14)

Hypertension (ICD-I10)	1193	74.3	1.00	-	1529	95.4	1.28	(1.19-1.38)***

Angina Pectoris (ICD-I20.9)	383	23.9	1.00	-	480	29.9	1.26	(1.10-1.43)**

Cardiac infarction (ICD-I21)	362	22.5	1.00	-	428	26.7	1.18	(1.03-1.36)*

Ischemic heart disease UNS (ICD-I25)	703	43.8	1.00	-	776	48.4	1.11	(1.00-1.22)*

Cardiac insufficiency (ICD-I50)	279	17.4	1.00	-	394	24.6	1.41	(1.22-1.65)***

Cerebral haemorrhage (ICD-I16.9)	17	1.1	1.00	-	35	2.2	2.06	(1.15-3.68)*

Stroke (ICD-I63)	187	11.6	1.00	-	236	14.7	1.26	(1.05-1.53)*

*p = 0.05,**p = 0.001,***p < 0.0001

**Table 3:** Cumulative incidence of cardiovascular diseases and cardiovascular risk factors (according to the diagnoses in ICD-10) and RR (risk ratios) for women aged 45-64 years (diagnosed 2002-2007) in the Twin cities.

	White-collar twin city				Blue-collar twin city			
	
	45-64 years (n = 16 011 inhabitants)				45-64 years (n = 16 031 inhabitants)			
	
	n	n/1000	RR	95% CI	n	n/1000	RR	95% CI
High Cholesterol (ICD-E-78)	450	28.1	1.00	-	504	31.4	1.12	(0.99-1.27)

Hypertension (ICD-I10)	946	59.1	1.00	-	1302	81.2	1.37	(1.27-1.49)***

Angina Pectoris (ICD-I20.9)	212	13.2	1.00	-	307	19.2	1.45	(1.22-1.72)***

Cardiac infarction (ICD-I21)	108	6.7	1.00	-	153	9.5	1.41	(1.11-1.81)*

Ischemic heart disease UNS (ICD-I25)	223	13.9	1.00	-	296	18.5	1.33	(1.12-1.58)**

Cardiac insufficiency (ICD-I50)	104	6.5	1.00	-	199	12.4	1.91	(1.51-2.42)***

Cerebral haemorrhage (ICD-I16.9)	8	0.5	1.00	-	20	1.2	2.50	(1.10-5.67)*

Stroke (ICD-I63)	116	7.2	1.00	-	154	9.6	1.33	(1.04-1.69)*

*p = 0.05,**p = 0.001,***p < 0.0001

The cumulative incidence of different cardiovascular diagnoses in elderly men and women (aged 65-79 years) is presented in table 4 and table 5. In addition, significantly higher relative rates were revealed among the elderly with regards to the studied cardiovascular diagnosis in the "blue-collar" twin city compared with the "white-collar" twin city. One exception was the diagnosis high cholesterol levels that were significantly more common among both males and females in the "white-collar" twin city. No difference in occurrence in elderly women was seen between the cities regarding myocardial infarction and stroke.

**Table 4:** Cumulative incidence of cardiovascular diseases and cardiovascular risk factors (according to the diagnoses in ICD-10) and RR (risk ratios) for men aged 65-79 years (diagnosed 2002-2007) in the twin cities.

	White-collar twin city				Blue-collar twin city			
	
	65-79 years (n = 6 892 inhabitants)				65-79 years (n = 6 695 inhabitants)			
	
	n	n/1000	RR	95% CI	n	n/1000	RR	95% CI
High Cholesterol (ICD-E-78)	832	121.7	1.00	-	656	98.0	0.82	(0.74-0.89)***

Hypertension (ICD-I10)	1486	215.6	1.00	-	1737	259.4	1.20	(1.13-1.28)***

Angina Pectoris (ICD-I20.9)	661	95.9	1.00	-	670	100.1	1.04	(0.94-1.16)

Cardiac infarction (ICD-I21)	537	77.9	1.00	-	598	89.3	1.15	(1.03-1.28)*

Ischemic heart disease UNS (ICD-I25)	1055	153.1	1.00	-	1161	173.4	1.13	(1.05-1.22)**

Cardiac insufficiency (ICD-I50)	750	108.8	1.00	-	806	120.4	1.11	(1.01-1.22)*

Cerebral haemorrhage (ICD-I16.9)	20	2.9	1.00	-	51	7.6	2.63	(1.57-4.40)***

Stroke (ICD-I63)	396	57.5	1.00	-	406	60.6	1.06	(0.92-1.21)

*p = 0.05,**p = 0.001,***p < 0.0001

**Table 5:** Cumulative incidence of cardiovascular diseases and cardiovascular risk factors (according to the diagnoses in ICD-10) and RR (risk ratios) for women aged 65-79 years (diagnosed 2002-2007) in the twin cities.

	White-collar twin city				Blue-collar twin city			
	
	65-79 years (n = 8 151 inhabitants)				65-79 years (n = 8 015 inhabitants)			
	
	n	n/1000	RR	95% CI	n	n/1000	RR	95% CI
High Cholesterol (ICD-E-78)	608	74.6	1.00	-	526	65.6	0.88	(0.79-0.99)*

Hypertension (ICD-I10)	1644	201.7	1.00	-	2026	252.8	1.25	(1.18-1.33)***

Angina Pectoris (ICD-I20.9)	548	67.2	1.00	-	616	76.9	1.14	(1.02-1.28)*

Cardiac infarction (ICD-I21)	392	48.1	1.00	-	391	48.8	1.01	(0.89-1.16)

Ischemic heart disease UNS (ICD-I25)	542	66.5	1.00	-	645	80.5	1.21	(1.08-1.36)**

Cardiac insufficiency (ICD-I50)	539	66.1	1.00	-	700	87.3	1.32	(1.19-1.47)***

Cerebral haemorrhage (ICD-I16.9)	16	2.0	1.00	-	42	5.2	2.67	(1.50-4.74)**

Stroke (ICD-I63)	316	38.8	1.00	-	348	43.4	1.12	(0.97-1.30)

*p = 0.05,**p = 0.001,***p < 0.0001

## Discussion

This study revealed that the risk for cardiovascular morbidity and mortality is very different between the population in a "blue-collar" and a "white-collar" city. These differences seem to be profound and stable over time. The two studied cities are quite comparable and referred to as twin cities, although historically they represent different social environments, a "blue-collar" and a "white-collar" city. By this study design we want to uncover the relative importance of the social environment for cardiovascular risks in a defined population.

The strength of this study is that it is based on computerised data files (HCR) of all inhabitants linked by birth date and gender. The same personal code is used for all visits and diagnoses in HCR. An individual can thus be followed retrospectively or prospectively through the health care system. The health care institution where the patient was diagnosed represents all health care levels, primary care, outpatient hospital care, and/or inpatients hospital care. General limitations in register data is that misclassifications do occur, including cases that are not recorded because they are overlooked or given incorrect clinical codes. There could also potentially be some referral bias. However, according to The National Board of Health and Welfare, analysis of the quality and content of the Swedish Hospital Discharge Register indicates up to 98% coverage of main diagnostic codes in in-patient care in this region [31]. Validation of HCR and other comparable administrative health care data registers has shown high specificity in registers covering all type of health care [33-35]. Therefore it can be assumed that HCR data will show high specificity. When comparing geographically close cities one could always assume that there are some migration between the cities. However, it is unlikely that this population migration is health related so it could introduce a health selection bias in the study. The diagnose comparisons between the two cities were not age-adjusted but stratified for age. The age strata might appear broad (45-64; 65-79 years) and age is a strong predictor of cardiovascular diseases. However, the age-distribution in these two cities is quite equal leaving only a minimal risk of possible confounding by age.

A close connection between socio-economic factors and cardiovascular morbidity has long been known [36]. Low socio-economic status is associated with an increased risk for cardiovascular disease (CVD) in both men and women [6,8,19]. This was confirmed in a study in northern Sweden where both higher incidence and higher mortality in myocardial infarction and stroke were found amongst blue-collar workers compared to subjects in non-manual occupations [37]. Major differences in cardiovascular risk profiles have also been found between skilled and unskilled blue-collar workers [38].

Individual behaviour and lifestyle and thereby socio-economic factors are of importance when analysing cardiovascular risk determinants [26,39,40]. The highest attained educational level is a good proxy for socio-economic status. Educational level is further the strongest component in the socio-economic concept [30]. Educational level reflects not only living conditions, but also attitudes and health behaviour in general. Differences in educational levels of the population in the studied twin cities might contribute to the difference in risk of cardiovascular disease. In a city with a mix of more highly educated people (the "white-collar" twin city) we will probably find different health behaviour, life style, consumption patterns and dietary habits than in a city with a less educated people (the "blue-collar" twin city), thus leading to differences in the risk of cardiovascular disease between these cities.

Associations between neighbourhood socioeconomic characteristics in the population and hypertension risk have in other studies found to be mediated by differences in body mass index [25]. This indicates that dietary habits are closely connected to educational level. Similarly, research suggests that people who have obese individuals among their closest friends also tend to have a risk of becoming obese over time [41]. Health behaviour tends to form in the micro-systems and close social relationships within a society. Societies with more educated people, even in the social micro-systems, thereby tend to exhibit different life style and cardiovascular risks than in communities with more lower educated people. Different social norms are developed in cultural groups and subgroups in a society. These norms include explicit as well as implicit rules for what attitudes and behaviours are appropriate in this specific group. Individuals who do not follow the rules might be excluded from the group. These mechanisms could be an explanation why a community-based programme might be more successful than when individual programmes are offered [42]. In the long run, a community-based programme provides the possibility to influence the social norm defining the appropriate way to live in the specific group.

In general, the health situation with regard to morbidity, mortality and self-reported health in the "blue-collar" twin city is in general notably worse than in the "white-collar" twin city. These differences have not diminished over time from the late 1970s and onwards.

There are noticeable socio-economic and socio-cultural differences between the two studied cities that might contribute to the explanation of the cardiovascular differences found in this study. Although the traditional industrial worker hardly exists today, not even in the "blue-collar" city, the older generation in particular still identifies with that role. In many respects, the "blue-collar" twin city has gradually lost its leading role in the region during recent decades in terms of population, political weight, strategic investments etc. Widespread factory close-downs in the 1960s and 1970s resulted in high unemployment in the "blue-collar" twin city. In addition, relatively poor working conditions as well as the powerlessness of people in this situation are detrimental for health. Until the last 10-15 years the "blue-collar" twin city was "a town of sorrow", with an old structure and a history of closed factories. All these factors might have an impact on the risk for cardiovascular disease in the population. However, trends that could have positive effects on health have also emerged in the last decade since the university has also established a university campus in the "blue-collar" twin city. Today, many social structures in the "blue-collar" twin city are approaching the structures of the neighbouring "white-collar" twin city, but public health differences seem to prevail.

The "white-collar" twin city has experienced a strong economical and population growth throughout the 20th century especially in the post-war period. This is especially due to the establishment of an aerospace industry with guaranteed governmental orders of airplane production, which created a demand for engineers and thereby manifested the "white-collar" city as a city of high technology. This city managed to turn a precarious situation in the 1960's, mainly through the establishment of the Technical University College and eventually a fully-fledged university. A successful collaboration between the University and the County Council also led to the establishment of a medical faculty in the 1980s. The economic and political success of a city is probably a health-promoting factor in itself. A differentiated industry leading to a diverse labour market in the "white-collar" municipality is certainly even more important. The transformation from an industrial society to technological information society was faster than in the neighbouring "blue-collar" twin city. However, the other side of the coin reveals a rising income gap and increasing ethnical segregation in the "white-collar" twin city in the last decade.

It is possible to change the risk of cardiovascular disease not only on the individual level but also in a population and in a whole community. There are some good international examples of programmes how community-based intervention actually reduces the risk of cardiovascular disease in a community. Since the early 1970s, community-based programmes have shown to be effective in the field of cardiovascular disease prevention. A major intervention program, The North Karelian project, was launched in eastern Finland, where morbidity and mortality incidence was among the highest in Europe [43,44]. The idea was to change the general risk-related ways of living of the whole population through community-based actions that included health education and medical check-ups, as well as work with a range of organisations, such as local non-governmental organisations, political decision-makers and the private industrial sector. The results of the project is quite positive but must be evaluated against a background of a general decline in cardiovascular diseases in the society.

However, these projects did not take into account the voices of ordinary people living in the area of prevention programmes. Ways that people who suffers from heart diseases understand and make sense of their disease has been reported in medical anthropological research [45]. Another good preventive example is the "Västerbotten Intervention Program" aiming to reduce cardiovascular risks in northern Sweden [46-48]. Using an upstream perspective through collaboration between politicians, healthcare providers, primary care and the public, a stable community-based intervention program was launched in the mid-1980s. The results are very encouraging, as a steady reduction of cardiovascular morbidity and mortality in this population has been documented [49,50].

## Conclusions

There are profound differences in cardiovascular morbidity and mortality between the studied twin cities representing two close but different social environments. These differences seem not to diminish but to be stable over time, although both cities have undergone social and economic changes from industrial to post-industrial cities. Health behaviours and lifestyles seem to prevail in both of these social environments casting a shadow of higher risk of cardiovascular disease in the population of the "blue-collar" city. This data give strong public health implications with an upstream approach to initiate a long-term community intervention programme in the "blue-collar" twin city.

## Competing interests

The authors declare that they have no competing interests.

## Authors' contributions

CW, A-KJ, TF participated in the study design and coordination and completed the data collection. CW, A-KJ, TF drafted the manuscript. All authors contributed to analysis and interpretation of data, and read and approved the final manuscript.
